# Physicochemical Characterization and Evaluation of the Suspending Properties of *Boswellia papyrifera* Gum in Metronidazole Benzoate Suspension

**DOI:** 10.1155/2024/8899359

**Published:** 2024-09-20

**Authors:** Gebremariam Woldu, Tsegay Brhane, Gebre Teklemariam Demoz

**Affiliations:** Department of Pharmacy College of Health Sciences Aksum University, Aksum, Ethiopia

**Keywords:** gum, sedimentation volume, suspending agent, viscosity

## Abstract

**Background:** Currently, many natural gums are extensively utilized as suspending agents in the formulation of pharmaceutical suspensions. They are easily available, nontoxic, biodegradable, and cost-effective to be used as pharmaceutical excipients.

**Objective:** The present study was aimed at physicochemical characterization and evaluation of the suspending capacity of *Boswellia papyrifera* gum (BPG) in comparison with sodium carboxy methyl cellulose (SCMC) and tragacanth gum (TG).

**Methods:** The extracted and powdered BPG was subjected to physicochemical properties such as micromeritics, solubility, swelling power, ash value, moisture content, conductivity, pH, and apparent viscosity using standard methods. Metronidazole benzoate suspensions were formulated using various concentrations of BPG, SCMC, and TG (1%–5% *w*/*v*). The apparent viscosity, flow rate, sedimentation volume, redispersibility number, pH, and drug content were studied as assessment parameters.

**Results:** The micromeritic studies revealed that BPG exhibited good flow properties. There was also a significant increase in solubility and swelling power of the gum as a function of temperature. The gum had 2.78% ash value and 4.32% moisture content. Conductivity and apparent viscosity of the gum were found to be increased with concentration (*p* < 0.05). However, the apparent viscosity of BPG was decreased with shear rate (*p* < 0.05), rendering a pseudoplastic flow property of the gum, which is an ideal characteristic of suspending agents. The suspending capacity of the BPG was found to be comparable to SCMC, but higher than TG. Thus, it can be concluded that BPG could be used as the best alternative to natural and synthetic suspending agents.

## 1. Introduction

**Figure 1:**
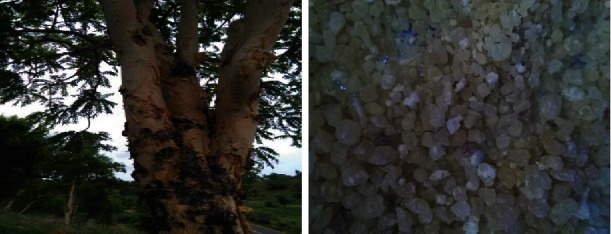
*Boswellia papyrifera*, located in the western zone of Tigray [8].

Pharmaceutical suspensions are heterogeneous type of liquid dosage forms that consists of the internal phase (insoluble solid particle) and external phase (liquid phase) [1]. As a result of their sizes, the active pharmaceutical ingredient (insoluble solid particles) within the pharmaceutical suspensions has high sedimentation rate [2]. As a consequence, pharmaceutical suspensions are physically unstable dosage forms [3]. However, in a stable pharmaceutical suspension, the suspended material should not settle too rapidly, the settled particles must not form a hard mass, and the suspension must not be too viscous to pour freely from the container. This is attained by increasing the viscosity of the liquid vehicle, thereby reducing the settling solid particles in accordance with the Stokes law [4]. Hence, in order to achieve such kind of quality, the incorporating of suspending agents into the formulation of suspensions has a substantial effect [5]. The study done in Ethiopia showed that gum obtained from *Acacia polyacantha* had a good suspending capacity compared to acacia and sodium carboxy methyl cellulose (SCMC) in metronidazole benzoate suspension [6]. Due to the fact that Ethiopia has many *Boswellia* species (Burseraceae families) [7], it is very important to investigate the untapped natural resources of the country for their potential utility as a safe, effective, and cheaper suspending agents. This is not only to enhance the availability of alternative pharmaceutical excipients but also to save foreign expenditures, create additional income to local farmers, create job opportunity, and give up economic development. Thus, the present work is aimed at comparing the suspending ability of *Boswellia papyrifera* gum (BPG) (Figure 1) with sodium carboxyl methylcellulose and tragacanth gum (TG).

## 2. Materials and Methods

### 2.1. Materials

#### 2.1.1. Materials

Materials and chemicals used in this study were sourced from different companies. They include metronidazole benzoate (Hubei Hungyanphar), TG (Unival, Germany), Na-CMC, methyl paraben, propyl paraben (Zhejiang, China), propylene glycol (Unival, Germany), hydrochloric acid (HCl) (Zhejiang, China), polysorbate 80, saccharin sodium, sucrose (Dubai), citric acid, sorbitol 70% solution, distilled water, and ethanol. All chemicals utilized in this experiment were found to be analytical grade.

#### 2.1.2. Equipment

The following equipment was utilized in this work: weighing balance, mortar and pestle, water bath, conductometry (SevenCompact), sieve shaker, oven, magnetic stirrer, dissolution apparatus (Pharma Test), UV spectrophotometer (Shimadzu Corporation), pH meter (Nickel Electro Ltd.), and viscometer (HAAKE Viscotester 6 Plus).

#### 2.1.3. Plant Collection and Identification

The crude plant material (*B. papyrifera* gum) was collected from Humera, western zone of Tigray, where the plant grows naturally. The collected plant material was authenticated by the taxonomist in the Department of Biology, Addis Ababa University.

### 2.2. Methods

#### 2.2.1. Gum Extraction and Percentage Yield

The gum collected from *B. papyrifera* was dried in a hot oven at 60°C for 24 h, milled in a blender, and sieved to a mesh size of 224 *μ*m. In order to extract the pure gum portion, 100 g of the powder was stirred with 500 mL of ethanol 90% for 2 h. The ethanolic slurry was filtered via Whatman No. 1 filter paper. The residue was washed with 100 mL of ethanol 90% *v*/*v* twice, and then, the residue was dried at 40°C for 12 h in a hot oven. After drying, the powder was dissolved in 500 mL distilled water at room temperature and then filtered through a muslin cloth. Thereafter, the gum was precipitated from the slurry using ethanol (90% *v*/*v*) in 1:2 ratio, filtered through a muslin cloth, and the precipitate was dried in an oven at 40°C for 48 h. The dried extract was pulverized using a blender to fine the particles, sieved through a 224 *μ*m sieve, and stored in an air-tight container. The percentage yield of the gum was determined by dividing the weight of the dried gum after extraction by the weight of the fresh gum [9].

#### 2.2.2. Physicochemical Characterization of the Gum

The gum was powdered finely using mortar and pestle and sieved to a mesh size of 224 *μ*m, and characterization of the gum was performed using the following parameters.

#### 2.2.3. Determination of True Density

True density is defined as the mass of a particle without any void space within the particle. The true density of 5 g BPG was determined by the liquid displacement method using toluene as the immersion fluid with a known volume and accurately weighed pycnometer [10].

(1) $$\begin{array}{c} \text{true density}=\frac{\text{weight of the sample}}{\left[\left(\text{weight of fluid}-\text{filled Pycnometer}+\text{weight of fluid}-\text{filled Pycnometer plus the sample}\right)-\text{weight of the sample}\right]\times \text{density of the fluid}} \end{array}$$

#### 2.2.4. Determination of Bulk Density and Tap Density

To determine the bulk density of the gum, 100 g weight of BPG was placed in a 500-mL clean and dry measuring cylinder. The volume occupied by the sample without tapping was noted as bulk volume. Whereas in order to determine the tapped density of the gum, tapped volume was recorded by tapping the gum powder using Tap Density Analyzer (ERWEKA, Germany) [11]. This procedure was performed three times, and the bulk density (Equation (2)) and tap density (Equation (3)) of the gum were calculated.

(2) $$\begin{array}{c} \text{Bulk density}=\frac{\text{weight of the sample powder }\left(\text{g}\right)\;}{\text{bulk volume }\left(\text{mL}\right)} \end{array}$$

(3) $$\begin{array}{c} \text{Tapped density}=\frac{\text{weight of the sample powder }\left(\text{g}\right)}{\text{tapped volume }\left(\text{mL}\right)} \end{array}$$

#### 2.2.5. Determination of Density-Related Properties

The Carr's index and Hausner's ratio of gum have been used as an indirect method of flowability measurement and determined using the following Equation (12).

(4) $$\begin{array}{c} \text{Car}{\text{r}}^{\text{'}}\text{s index }\left(\%\right)=\frac{\text{tapped density}–\text{bulk density}}{\text{tapped density}}\times 100 \end{array}$$

(5) $$\begin{array}{c} \text{Hausner ratio}=\frac{\text{tapped density}}{\text{bulk density}} \end{array}$$

#### 2.2.6. Angle of Repose

Funnel method was used to determine the angle of repose of the gum [12]. The funnel was made permanent by a retort stand; the base of the orifice is 10 cm over the table surface. Finally, the funnel was filled with a gum powder and allowed to pour on a graph paper placed on the table. The average radius (*r*) and height (*h*) of the powder piles formed on the table were recorded. The experiment was repeated three times, and the angle of repose (*θ*) was calculated (Equation (6)).

(6) $$\begin{array}{c} \text{Angle of repose}\left(\theta \right)=\tan^{-1}\left(\frac{h}{r}\right) \end{array}$$

#### 2.2.7. Moisture Content

To determine the moisture content of the gum, 1 g of the gum was transferred in to a dried evaporating dish with known weight. It was heated at 105°C in hot oven for 4 h. The dish was then removed and allowed to cool for 30 min in a desecrator. Then, the dish was weighed as quickly as possible, and the moisture content of the sample was calculated as the weight difference of the sample before and after drying in the oven [13].

(7) $$\begin{array}{c} \text{Moisture content }\left(\%\right)=\frac{\text{weights of the gum before drying}-\text{weights of the gum after drying}}{\text{weights of the gum before drying}}\times 100\% \end{array}$$

#### 2.2.8. Ash Value

Two grams of the gum was weighed accurately in a previously tared crucible and spread in an even layer within the crucible and heated in a furnace at 450°C for 6 h until becoming white; the crucible was then taken out of the furnace, cooled in a desiccator, and weighed, and the ash value (percent) of the gum was calculated [14].

(8) $$\begin{array}{c} \text{Ash value}=\frac{\text{weight of ash formed }}{\text{initial weight of the gum}}\times 100 \end{array}$$

#### 2.2.9. Solubility and Swelling Power

The solubility and swelling power of the gum was determined by dispersing 1 g of the gum in 8 mL of distilled water in centrifuge tubes. The tubes were put in a thermostatically controlled water bath at a temperature of 10°C, 20°C, 30°C, 40°C, 50°C, and 60°C with shaking for 10 min. The dispersions were centrifuged at 3000 rpm for 15 min. The supernatant was collected in preweighed evaporating dish and dried in hot oven at 105°C for 3 h. The residues obtained after drying the supernatant represent the amount of soluble gum, whereas the sediment is the swelling power of the gum [15].

(9) $$\begin{array}{c} \text{Solubility}=\frac{\text{mass of dried supernatant}}{\text{mass of the gum taken}}\times 100\% \end{array}$$

(10) $$\begin{array}{c} \text{Swelling power}=\frac{\text{mass of the the precipitated gum}}{\text{mass of the gum taken}-\text{mass of dried supernatant}} \end{array}$$

#### 2.2.10. Conductivity

Different masses of BPG powders were dispersed in distilled water to prepare 1, 2, 3, 4, and 5 (percent *w*/*v*) dispersions. The dispersions were stirred using a magnetic stirrer (50 rpm) for 2 h at room temperature and evaluated for conductivity using conductivity tester [16].

#### 2.2.11. pH

The pH of 1, 2, 3, 4, and 5 (percent *w*/*v*) BPG dispersion in distilled water was measured using a calibrated digital pH meter, and the study was conducted at room temperature [17].

#### 2.2.12. Apparent Viscosity

##### 2.2.12.1. Effect of Gum Concentration on Apparent Viscosity

To determine the effect of concentration on apparent viscosity, different weights of the gum powder (1, 2, 3, 4, and 5 g) were dispersed in 100 mL distilled water with continuous stirring. The dispersions were kept overnight at room temperature. The apparent viscosities of each dispersion were determined by a viscosity tester using Spindle Number 2 at shear rate of 20 rpm [18].

##### 2.2.12.2. Effect of Shear Rate on Apparent Viscosity

To measure the effect of shear rate on apparent viscosity, 3 g of the gum powder was dispersed in 100 mL of distilled water with continuous stirring. The dispersion was then kept overnight at room temperature. The apparent viscosity of the dispersion was determined by a viscosity tester using Spindle Number 2 at different shear rates (20, 30, 50, 60, 100, and 200 rpm) [18].

#### 2.2.13. Preparation of Metronidazole Benzoate Suspensions

The compositions of metronidazole benzoate suspensions are given in Table 1. TG, BPG, and SCMC containing formulations were used as suspending agents in suspension formulations. The formulations were grouped into three: FT (for TG), FB (for BPG), and FS (for SCMC) containing formulations. Each group has five formulations (FT1–FT5, FB1–FB5, and FS1–FS5). The letters FT, FB, and FS indicate the type of suspending agent, while the Numbers 1, 2, 3, 4, and 5 represent the concentrations of the suspending agents. To prepare the suspension, 46.5 mL of distilled water was transferred into a 250-mL flask and heated up to 95°C. Methyl paraben, propyl paraben, sucrose, saccharin, sodium, and citric acid were added, and the mixture was stirred for 2 h at 95°C with the help of a magnetic stirrer. The suspending agents were dispersed in distilled water and propylene glycol for 30 min and then mixed with the simple syrup. The dispersion containing metronidazole benzoate, Tween 80, and sorbitol solution was added to the vehicle and then stirred continuously until uniform dispersion was obtained [19].

### 2.3. Evaluation of Metronidazole Benzoate Suspensions

#### 2.3.1. Apparent Viscosity of the Suspensions

Viscosity has great importance for the stability and pourability of pharmaceutical suspensions. The apparent viscosities of the formulations containing BPG, SCMC, and TG as suspending agents were determined at room temperature at different shear rates (20, 30, 50, 60, 100, and 200 rpm) using viscosity tester (R2) [19]. All determinations were made in triplicate, and the results obtained were expressed as the mean values.

#### 2.3.2. Flow Rate of the Suspensions

The flow rate of the suspension was measured by the time required for 10 mL of the suspension to flow through a pipette [5].

(11) $$\begin{array}{c} F=\frac{VS}{T} \end{array}$$

where *F*, *Vs*, and *T* are the flow rate, volume of solution in the pipette (milliliters), and the time (seconds) required for the 10 mL suspension to totally elute out of the pipette, respectively.

#### 2.3.3. Sedimentation Volume of the Suspensions

Hundred milliliters of each formulation was derived to a 200-mL measuring cylinder and permitted to stand for a preset period of time at room temperature without shaking. The volume occupied by the solute was noted every day for 7 days and afterwards every week for 3 weeks successively [20]. The sedimentation volume (*F*) was calculated based on Equation (12), and the recorded results were averages of three determinations.

(12) $$\begin{array}{c} \text{Sedimentation volume}=\frac{\text{ultimate settling height }f\text{ the suspension}}{\text{original height of the suspension before settling}}\times 100\% \end{array}$$

##### 2.3.3.1. Effect of Electrolyte Concentration on Sedimentation Volume

To 100 mL of the suspension containing 3% of the suspending agents, various concentrations of NaCl such as 10^−4^, 10^−3^, 10^−2^, and 5 × 10^−2^ molar were mixed with the preparation independently, and the sedimentation volume of the individual formulations was recorded daily for seven successive days [8, 21]. The results recorded were averages of three determinations.

##### 2.3.3.2. Effect of pH on Sedimentation Volume

The effect of pH on the suspension was examined by treating 100 mL of the suspension containing 3% of the suspending agents with 0.1 N HCl and 0.1 N NaOH (at pH values of 2, 6.5, 8, and 10). Each preparation was poured into measuring cylinder, and the sedimentation volume was noted every day for seven succeeding days [8].

#### 2.3.4. Redispersibility Rate of Suspensions

A constant volume of each formulation (100 mL) was maintained and stored for various time intervals (1 week and 4 weeks) in a measuring cylinder for 1 month at room temperature. The measuring cylinder was shaking upside down manually, and the number of times the cylinder was sharked until the sediment was uniformly redispersed was recorded [8].

#### 2.3.5. pH of the Suspensions

The pH of a particular suspension was determined weekly for 4 weeks by using a pH meter [22].

#### 2.3.6. Assay of the Suspension

The active drug within the formulations was determined by UV visible spectrophotometer. Ten milliliters of the suspension from each formulation was transferred into the volumetric flask (100 mL), and the volume was made up to 100 mL with 0.1 N HCl. Two milliliters from each formulation was withdrawn from the flask and added into another volumetric flask (200 mL), and the volume was made up to 200 mL with 0.1 N HCl and filtered. Then, using 0.1 N HCl as a blank, the absorbance was measured using UV visible spectrophotometer at *λ* max of 242 nm [6].

#### 2.3.7. Dissolution Profile of the Suspensions

Dissolution (in vitro) profiles of the suspensions were performed in a dissolution tester (paddle method) (Pharma Test, Germany). The dissolution medium was 0.1 N HCl (900 mL) and maintained at 37 ± 0.5°C, and the paddle rotation speed was 50 rpm. From each suspension, 5 mL samples were withdrawn at 10, 20, 30, 40, 50, and 60 min. The collected samples were filtered and diluted to 200 mL using 0.1 N HCl. Finally, the absorbance of each formulation was measured using UV spectrophotometer at *λ* max of 242 nm by using 0.1 N HCl as a blank [23].

### 2.4. Statistical Analysis

Statistical analysis was carried out using analysis of variance (ANOVA) with Origin 8 (Origin Lab Corporation, United States) software. At 95% confidence interval, *p* values less than or equal to 0.05 were considered statistically significant. All data measured and reported in this study were average of a minimum of triplicate measurements, and the values are expressed as mean ± standard deviation (SD).

## 3. Results and Discussion

### 3.1. Percentage Yield

The percentage yield of the dried and powdered gum obtained after the extraction of *B. papyrifera* olio-gum resin was 77.67 ± 2.52%*w*/*w* which is much higher than the percentage yield of *Boswellia serrata* gum (6.96%) [24]. This might be attributed due the difference in species and the method of extraction. Hence, it is highly recommended to the pharmaceutical industry to utilize gum as a pharmaceutical excipient as the gum portion is the highest constituent in *B. papyrifera* olio-gum-resin complex.

### 3.2. Physicochemical Characterization of the Gum

#### 3.2.1. Density and Density-Related Properties of Gum

Density and density-related properties are significant parameters used to characterize the powder flow properties. True density, bulk density, tap density, Carr's index, Hausner's ratio, and angle of repose of the gum are shown in Table 2. The bulk and tap densities of the gum revealed that there was a marked reduction in the volume of the powders when subjected to tapping pressure. This indicated that the gum had good compaction properties [25]. Similar results were reported for *Moringa oleifera* Lam root extract with bulk density (0.29 g/mL) and tapped density (0.36 g/mL) [26]. Usually, Hausner's ratio (≤ 1.25) and Carr's index [5–7, 9–16, 27] represent for powders having good flow properties. Angle of repose (< 30°) usually points to a free-flowing material, while an angle of repose (≥ 40°) reveals poorly flowing material [28]. In this investigation, the values of Hausner's ratio, Carr's index (%), and angle of repose revealed that BPG possesses good flow properties. Hence, it can be deduced that BPG has demonstrated good flow characteristics and might have a potential use as an excipient in pharmaceutical dosage forms.

#### 3.2.2. Moisture Content

Moisture content of excipients can influence the powder flowability and stability of the finished pharmaceutical product [29]. The moisture content of BPG powder was 4.32%, which is within the pharmacopoeial specification [30]. The value of moisture content was lower to those reported in the literature [31], for example, flaxseed gum (8.3%), guar gum (11.7%), and xanthan gum (14.3%), respectively. Hence, the gum exhibits good flow properties and inferred to have a stable finished product. This might be due to nonhygroscopic nature of the gum.

#### 3.2.3. Ash Value

Ash value is used to measure the contamination and adulteration of the gum with inorganic materials [32]. Ash value of the gum was found to be 2.78 which is comparable with the study conducted on *B. serrata* gum [25]. However, it is lower than *Abelmoschus esculentus* leaf gum which had an ash values of 19.70 [33]. The low ash value obtained in this study exhibits low levels of contamination and adulteration of the gum. Therefore, BPG is a good candidate to be utilized as a pharmaceutical excipient.

#### 3.2.4. Solubility and Swelling Power

Solubility of BPG in distilled water with respect to temperature is depicted in Table 3. As shown in the table, the solubility of the gum was increased significantly (*p* < 0.05) from 34.93%–96.19% with increased temperature from 25°C–85°C. The observed increase in solubility with temperature indicates that the heat given off in dissolving the gum is less than the heat required to break the gum apart [34]. The solubility profile of the gum was consistent with the findings reported elsewhere [35–37]. The swelling power of any natural gum depends upon its water retention capacity or water absorption capacity [29]. Swelling power of the gum was found to be increased significantly (*p* < 0.05) from 22.83–98.51 with increased temperature from 25°C–85°C (Table 3). This might be due to the entanglement of polysaccharide chains and the development of intramolecular and intermolecular hydrogen bonds between the polysaccharide and water [10]. The high swelling power of the gum may endow it to act as a suspending agent in pharmaceutical suspensions [38].

#### 3.2.5. Conductivity

The effect of gum concentration on conductivity is illustrated in Table 4. Accordingly, as the concentration of gum increased from 1% to 5%, the conductivity was also raised from 258.7 to 1324.3, correspondingly. The increment in conductivity with the increment in the concentration of gum might be due to the presence of different ions or mono- and divalent electrolytes in the gum [39]. Similar findings have been reported on the conductivity of *Opuntia cochenillifera* mucilage [40]. Gums having high concentrations of electrolytes exhibit a higher in their sedimentation volume [22]. Hence, the gum has excellent potential to be utilized as a suspending agent.

#### 3.2.6. pH

The results of pH at different concentrations are presented in Table 4. It was observed that BPG had a pH range of 4.87–5.40, which revealed that the gum is slightly acidic in nature. This acidic nature indicates that the gum contains uronic acids in its structure [41]. The pH values are comparable to those reported for *Cola acuminata* gum having a pH value of 5.37 [2]. Natural polymers having lower pH values exhibited a higher in their viscosity [42]. Thus, gum has an importance for its potential use as a suspending agent.

#### 3.2.7. Apparent Viscosity

##### 3.2.7.1. Effect of Concentration on Apparent Viscosity

The effect of concentration on the apparent viscosity is depicted in Table 4. From this table, the apparent viscosity of BPG dispersions was increased significantly (*p* < 0.05) with increasing concentration of gum. This might be associated with the strengthening of the intramolecular interaction on increasing the concentrations of the gums [43]. Similar results were reported on neem gum [44], carboxy methylated okra gum [32], *Sida acuta* gum [45], and welan gum [46]. The increment in viscosity concentration indicates that BPG can be used as a viscosity imparting agent in suspension formulations. The high viscosity of the polymer suggests application as a suspending agent, stabilizer, or thickener [47].

##### 3.2.7.2. Effect of Shear Rate on Apparent Viscosity

**Figure 2:**
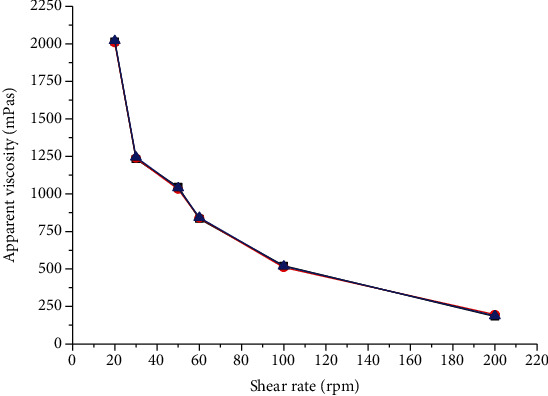
Apparent viscosity (millipascal second) of BPG at different shear rates.

Viscosity affects the ease of administration with which a suspension is withdrawn from its container. The less viscous suspension tends to pour more easily than the more viscous ones. Many fluids may change in their apparent viscosity with a change in shear rate. In this study, the apparent viscosity of 3 g BPG dispersion was decreased as the speed of rotation increases from 20 to 200 rpm (Figure 2). This indicated that the gum had a pseudoplastic flow property, which might be due to shear-thinning behavior of the gum [48]. Therefore, it can be suggested that BPG has the potential to be used as a suspending agent.

### 3.3. Evaluation of Metronidazole Benzoate Suspensions

#### 3.3.1. Apparent Viscosity of the Suspension

**Figure 3:**
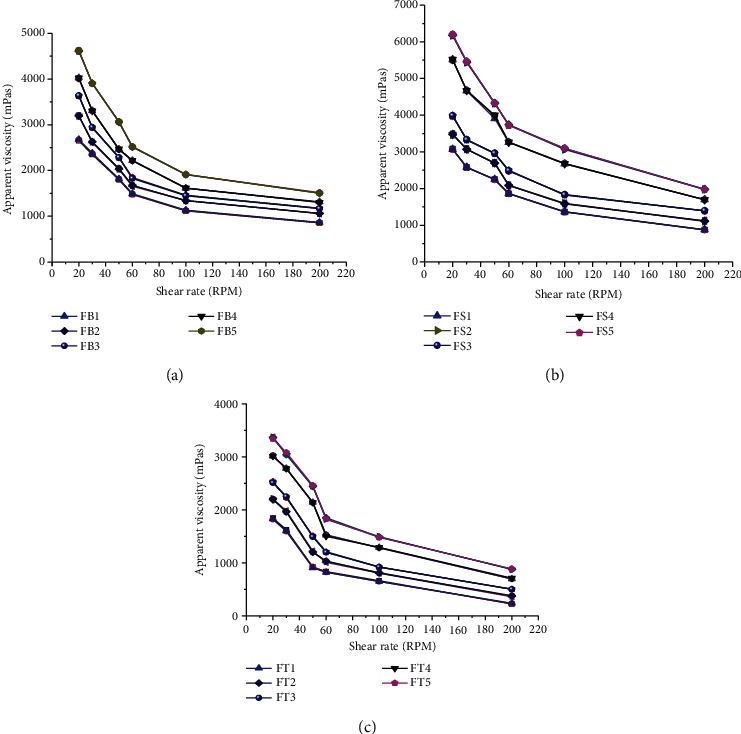
Apparent viscosities of suspensions (at different shear rates) prepared from (a) BPG, (b) SCMC, and (c) TG as suspending agents.

The pour ability and stability of a pharmaceutical suspension are fundamentally determined by its apparent viscosity [49]. The apparent viscosities of metronidazole benzoate suspensions prepared with BPG, SCMC, and TG are shown in Figure 3. From the figure, the apparent viscosities of all formulations were found to be increased significantly (*p* < 0.05) with concentration. This might be due to the increment in strength of the molecule–molecule interaction and the corresponding reduction in molecule–solvent interaction [50]. This is consistent with the investigation on suspending properties of *Brachystegia eurycoma* gum made by [4]. As the apparent viscosity of the suspension increases, the dispersed phase settles at a slower rate and remains dispersed for a longer period of time that yields higher sedimentation volume [6, 51]. Hence, the increment in the viscosity of the gum is a positive effect in lowering the rate of settling and enhancing stability of the formulated suspensions [43]. On the other hand, the apparent viscosities of all formulations were found to be decreased with increasing in shear rate. This might be attributed to the fact that as shear rate increased, the individual particles of the aggregate lost their intermolecular forces, broke apart, and aligned in the direction of increased shears [52]. This signifies the shear thinning properties of the formulated suspensions in which minimum agitation is required for the suspensions to be easily redispersed [53]. This is a desirable property of a good suspending agent. Generally, the differences in apparent viscosities of the formulated suspensions were significant (*p* < 0.05) and found in the order of SCMC > BPG > TG at the same concentrations and shear rates. These differences in viscosity among those suspending agents have an insinuation on the flow rate, sedimentation volume, rate of redispersibility, and dissolution rate of the suspension.

#### 3.3.2. Flow Rate of the Suspension

Flow rate in the pharmaceutical suspension is significant to determine the extent of pour ability of the formulated suspension from its container during administration resulting a better dosing of the suspension [6]. In this study, the flow rates of the suspensions were found to be varied with the concentration and type of suspending agents (Table 5). In all suspending agents, the flow rates of the suspensions were found to be decreased when the concentrations of suspending agents incorporated into the formulation were increased. This may be explained by the increment in viscosity of the products [54]. Similar results were obtained when *Corchorus olitorius* gum was evaluated for its suspending property [52]. In all batches of the formulations, the flow rates of the suspensions were found in the order of TG > BPG > SCMC. All suspension formulations were pourable except those containing SCMC at concentrations of 4% and 5% *w*/*v* due to their high viscosity.

#### 3.3.3. Sedimentation Volume of the Suspension

**Figure 4:**
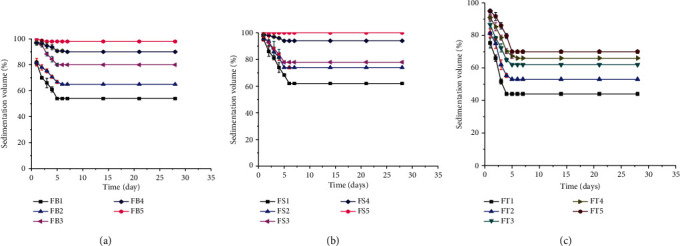
Sedimentation volumes of metronidazole benzoate suspensions containing (a) BPG, (b) SCMC, and (c) TG at different concentrations.

It is clearly recognized that the better suspending agent is an indication of lower sedimentation rate and once more higher in sedimentation volume. In this study, the sedimentation volumes of the suspensions based on the types of suspending agents, concentration, and time are given in Figure 4. ANOVA showed that there was a significant difference (*p* < 0.05) in sedimentation volume within the formulated suspensions containing different suspending agents. At lower concentration (1 and 2), BPG showed lower sedimentation volume than SCMC containing suspensions. At concentrations of 3, 4, and 5, both BPG and SCMC had comparable results in their sedimentation volume. This might be due to the presence of metallic contents within the gum that involved in some level of flocculation with the suspended drug particles [54]. In all concentrations of the suspending agents, formulations that contained TG as the suspending agent had the least in its sedimentation volume. This may be due to the low viscosity of the suspensions which allowed free movement of the suspended particles. High sedimentation volume is a signal that the bonding and interparticle attraction were loose and not strong enough to form a hard cake throughout the study period [51]. The results also showed that the sedimentation volumes of all suspensions prepared with BPG, SCMC, and TG were increased significantly (*p* < 0.05) when the concentrations of the suspending agents were increased. This might be due to a rise in the viscosity of the suspensions with the increment in the concentrations of the suspending agents that led to a reduction in the rate of settling of the drug particles [55]. Conversely, the values were decreased significantly (*p* < 0.05) with the time of storage in all batches of suspensions but remained constant after Day 5. This might be due to the effect of gravity on the suspended dispersed solute particles that eventually settles down with time [56]. Similar result was obtained when *A. esculentus* gum was evaluated for its suspending property in comparison with tragacanth and sodium CMC [33].

##### 3.3.3.1. Effect of Electrolyte Concentration on Sedimentation Volume

**Figure 5:**
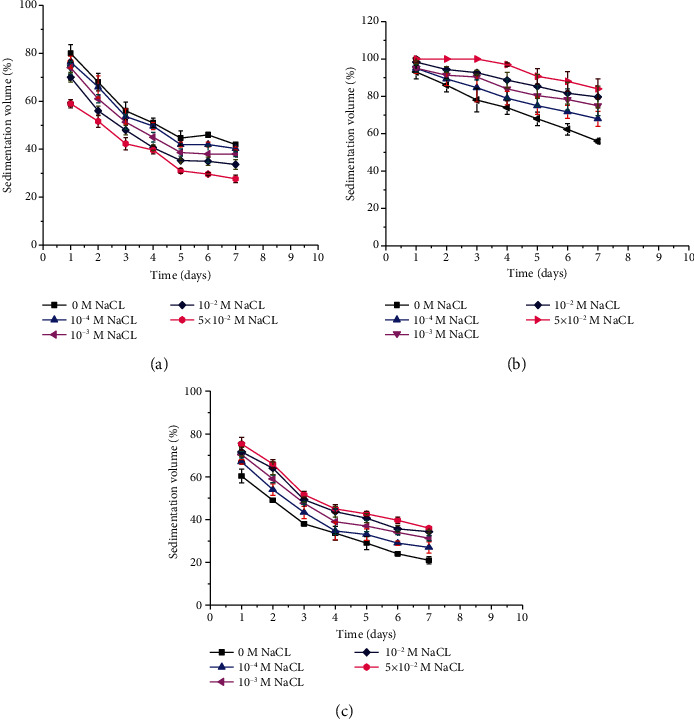
Effect of different NaCl concentrations on sedimentation volume of metronidazole benzoate suspensions prepared using 3% (a) BPG, (b) SCMC, and (c) TG as suspending agents.

The sedimentation volumes (percent) of metronidazole benzoate suspensions containing 3% of BPG, SCMC, and TG were found to be decreased significantly (*p* < 0.05) with the time of storage in all concentrations of NaCl (Figure 5). Formulations that had SCMC and TG as suspending agents have shown a continuous increment in their sedimentation volumes (percent) on increasing NaCl concentrations. This might be due to the flocculation of suspended particles via zeta potential reduction of the system [54]. However, BPG containing formulations have shown a decrease in their sedimentation volume (percent) with NaCl concentrations. This might be due to the compression of the extra double layer caused by increased ionic strength and formation of low rheological activity solution [43, 57]. Hence, the concomitant use of electrolytes as flocculating agent in formulations with BPG was found to be valueless, but with SCMC and TG, it is recommendable as the increment in sedimentation volume (percent) is significantly higher compared to the control.

##### 3.3.3.2. Effect of pH on Sedimentation Volume

**Figure 6:**
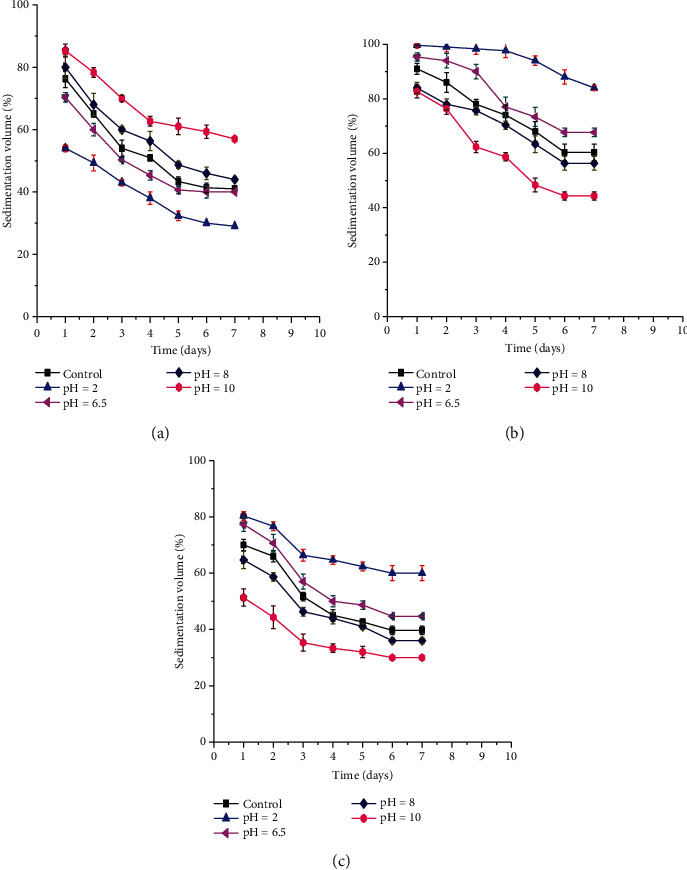
Effect of different pH on sedimentation volume of metronidazole benzoate suspensions prepared by 3% BPG, SCMC, and TG as a suspending agent.

The effects of pH (2, 6.5, 8, and 10) on the sedimentation volume of formulations containing 3% BPG, SCMC, and TG are depicted in Figure 6. In this study, formulations which had BPG, SCMC, and TG as suspending agents decreased their sedimentation volume throughout 7-day storage. The sedimentation volumes for BPG containing formulations were increased significantly (*p* < 0.05) with an increase in their pH values while decreased for TG and SCMC containing formulations. This might be due to the increment in the viscosity of the suspension containing BPG and the decrement in the viscosity of TG and SCMC containing suspensions with alkaline pH [58].

#### 3.3.4. Redispersibility of Suspensions

Pharmaceutical suspensions can produce sediment overtime due to gravitational forces. Hence, the sediments that are easily redispersed with minimal agitation are desirable to ensure a uniform dose of medicament after shaking [48, 59]. Pharmaceutical suspensions require the use of suspending agents in order to deliver a uniform dose of the active ingredient [60]. The number of inversions required to redisperse the sediment in all formulated suspensions with respect to time, concentration, and type of suspending agents is shown in Table 6. The numbers of inversions of the suspensions were increased significantly (*p* < 0.05) with storage time. More inversions were needed to redisperse the suspensions stored in a month than the corresponding suspensions stored in a week. The results also revealed that suspensions prepared with higher concentrations of BPG and SCMC were easier to redisperse than the corresponding lower concentrations. This might be attributed to the reduction of interparticle attraction which results loosely packed particles together, thereby making redispersion easier [2]. However, suspensions prepared with higher concentrations of TG were difficult to redisperse than the analogous lower concentrations. This might be due to the deflocculated nature of the system [61]. At the same time and concentration, the numbers of inversions to redisperse the suspensions prepared with BPG were significantly (*p* < 0.05) lower than those prepared with SCMC and TG suspensions. A lower number of inversions shows that the particles are in loose aggregates and can easily be redispersed by small agitation [2]. Hence, it was easier to redisperse suspensions containing BPG than those with SCMC and TG suspensions. This is an important prerequisite to have good and stable pharmaceutical suspensions [62].

#### 3.3.5. pH and Assay

Measurement of acidity or alkalinity of an excipient is an important parameter in determining its suitability in different formulations since the stability and physiological activity of most preparations depends on pH. From the table below, the pH and drug content of the suspensions are within the acceptable limits [63]. The pH values of the formulated suspensions were found to be within the slightly acidic to neutral pH range (Tables 7, 8, and 9). This implies that they would not cause any damage to the gastrointestinal tract after oral administration [53]. Hence, in liquid delivery systems, they are expected to be compatible with active pharmaceutical ingredients that have pH values within this range [64].

#### 3.3.6. Dissolution Profile of the Suspensions

**Figure 7:**
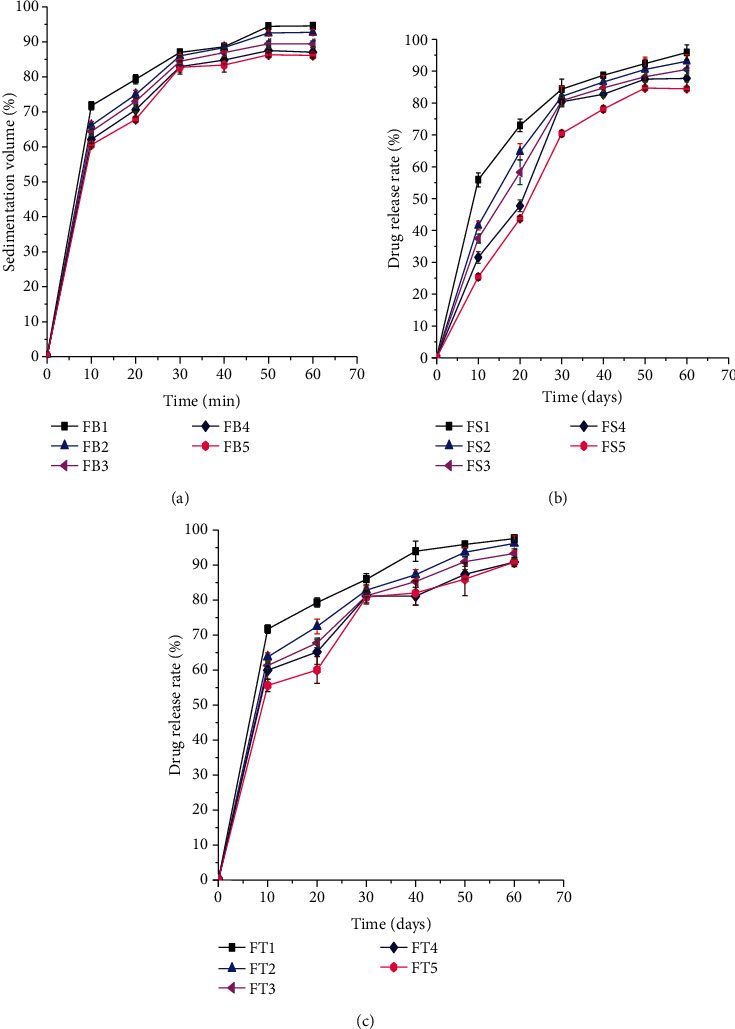
Drug release rate of all suspension formulations that have suspending agents: (a) BPG, (b) SCMC, and (c) TG.

The drug release profiles of metronidazole benzoate suspensions containing different concentrations of BPG, SCMC, and TG are displayed in Figure 7. The results showed that the amount of metronidazole benzoate released was increased significantly (*p* < 0.05) with respect to time for all formulations. The result showed that FB1, FB2, and FT1 released more than 85% of the metronidazole benzoate within 30 min, whereas FB3, FS1, FS2, FT2, and FT3 within 40 min. FB4, FB5, FS3, FS4, FT4, and FT5 attained the limit within 50 min. FS5 attained the limit within 60 min. Therefore, all formulations prepared with BPG, SCMC, and TG as a suspending agent released the drug within the USP acceptance range.

## 4. Conclusions

The powder-related properties of BPG exhibited good flow properties. The gum had 2.78% ash value and 4.32% moisture content. There was also a significant increase in solubility and swelling power of the gum as a function of temperature. Conductivity and apparent viscosity of the gum were found to be increased with concentration (*p* < 0.05). However, the apparent viscosity of BPG was found to be decreased with the increment of shear rate (*p* < 0.05), rendering a pseudoplastic flow property of the gum which is an ideal characteristic of suspending agent. The formulations of metronidazole benzoate suspensions containing BPG, SCMC, and TG as suspending agents exhibited pseudoplastic flow properties. The apparent viscosity and flow rate were in the order of SCMC > BPG > TG and TG > BPG > SCMC, respectively. At the same suspending agent concentration, the sedimentation volume (percent) of the suspensions containing BPG and SCMC was higher than TG containing suspensions. Moreover, BPG-based suspensions were found to be easier to redisperse than SCMC- and TG-based suspensions. Regarding pH, the drug content and dissolution rate of all formulations were within the acceptable limit. Thus, based on the above evaluation parameters, the suspending capacity of BPG was *higher* than TG and comparable with SCMC. Therefore, BPG could have a potential to be utilized as a suspending agent in pharmaceutical suspensions.

## Figures and Tables

**Table 1:** Formulation ingredients of metronidazole benzoate suspension.

**Formulation ingredients**	**Composition**
Metronidazole benzoate	6.43 (% *w*/*v*)
Suspending agents^a^	1, 2, 3, 4, and 5% (*w*/*v*)
Methyl paraben	0.18 (% *w*/*v*)
Propyl paraben	0.02 (% *w*/*v*)
Propylene glycol	2 (% *v*/*v*)
Tween 80	0.05 (% *v*/*v*)
Sodium saccharine	0.07 (% *w*/*v*)
Sucrose	15 (% *w*/*v*)
Sorbitol (70%, *w*/*v*)	30 mL
Citric acid	0.096
Distilled water, *Qs*	100

^a^Represents SCMC (sodium carboxy methyl cellulose), TG (tragacanth gum), and BPG (Boswellia papyrifera gum).

**Table 2:** Density and density-related properties of *Boswellia papyrifera* gum.

**Characteristics**	**Results**
True density	0.95 ± 0.01
Bulk density	0.26 ± 0.03
Tapped density	0.32 ± 0.02
Carr's index	10.07 ± 0.03
Hausner's ratio	1.13 ± 0.02
Angle of repose	24.43 ± 0.01

*Note:* Values are presented as mean ± standard deviation; *n* = 3. Mean value is significant (*p* < 0.05).

**Table 3:** Solubility and swelling power of *Boswellia papyrifera* gum at different temperatures.

**Temperature (°C)**	**Solubility**	**Swelling power**
25	34.93 ± 0.57a	22.83 ± 0.17a
40	39.2 ± 0.56b	27.79 ± 0.22b
55	55.3 ± 0.12c	34.79 ± 0.42c
65	72.72 ± 0.33d	65.43 ± 0.66d
75	88.69 ± 0.26e	80.71 ± 0.11e
85	96.52 ± 0.25f	98.36 ± 0.75f

*Note:* Values are presented as mean ± standard deviation; *n* = 3. The letters a, b, c, d, e, and f indicate the significant difference (*p* < 0.05) in solubility and swelling power of *Boswellia papyrifera* gum at different temperatures.

**Table 4:** Apparent viscosity (20 rpm), conductivity, and pH of BPG at different concentrations (mean ± SD, *n* = 3).

**Concentration (% ** **w**/**v)**	**Conductivity (*μ*s/cm)**	**pH**	**Apparent viscosity (mPas)**
1	258.67 ± 1.15a	5.40 ± 0.01a	178 ± 3.60a
2	307.66 ± 0.57b	5.07 ± 0.00b	282 ± 2.00b
3	673.66 ± 0.57c	4.93 ± 0.02c	750 ± 3.60c
4	841.33 ± 4.55d	4.89 ± 0.00c	1440 ± 6.56d
5	1324.33 ± 6.16e	4.87 ± 0.01c	2014 ± 6.55e

*Note:* a, b, c, d, and e indicate significant differences (*p* < 0.05) in apparent viscosity, conductivity, and pH at different concentrations.

**Table 5:** Flow rate of suspension with different concentrations of BPG, SCMC, and TG containing formulations.

**Concentration (% ** **w**/**v)**	**Flow rate of the suspension**		
	**BPG containing formulation**	**SCMC containing formulation**	**TG containing formulation**
1	0.57 ± 0.02a	0.14 ± 0.02a	1.15 ± 0.03a
2	0.23 ± 0.01b	0.10 ± 0.01b	0.86 ± 0.04a,b
3	0.12 ± 0.02c	0.07 ± 0.01b	0.82 ± 0.02a,b
4	0.09 ± 0.01c,d	Intermediate flow	0.59 ± 0.03b,c
5	0.05 ± 0.03d	No flow	0.34 ± 0.01c

*Note:* a, b, c, and d indicates a significant difference (*p* < 0.05) in the flow rate of BPG, SCMC, and TG containing formulations at different concentrations; intermediate flow is when the suspension does not fully flow out of the pipette. No flow is when the suspension does not flow out of the pipette with gravitational force.

**Table 6:** Redispersibility number of metronidazole benzoate suspensions prepared by BPG, SCMC, and TG after a week and a month (mean ± SD).

**Conc.**	**Redispersibility number (cycles)**					
	**After 7 days**			**After 1 month**		
	**BPG**	**SCMC**	**TG**	**BPG**	**SCMC**	**TG**
1	5.3 ± 0.8a	14 ± 1.0a	6 ± 0.0a	9 ± 2.0a	17 ± 2.0a	8 ± 0.0a
2	3 ± 2.0a,b	10 ± 3.0a	7 ± 0.0a	6 ± 1.00a,b	14 ± 1.0a	11 ± 1.00b
3	2 ± 1.0b	3 ± 1.0b	8 ± 1.0a,b	4 ± 2.0b	5 ± 2.0b	12 ± 0.0b
4	1 ± 0.0b	INR	10 ± 2.0b	3 ± 1.0b	2 ± 1.0b	13 ± 1.0b
5	INR	INR	13 ± 1.0c	INR	INR	17 ± 2.0c

*Note:* a, b, and c show significance (*p* < 0.05) difference among formulations containing different concentrations of suspending agents; INR—there is no need to redisperse the suspension (no sediment is formed at the bottom of the container).

**Table 7:** pH and drug content of BPG containing metronidazole, Metronidazole benzoate suspensions.

**Conc.**	**BPG containing formulation**				
	**pH**				**Drug content (%)**
	**1st day**	**7th day**	**14th day**	**21th day**	**1st day**
1	5.72 ± 0.09a	5.34 ± 0.2a,b	5.30 ± 0.04b	5.30 ± 0.04b	100.33 ± 0.71a
2	5.68 ± 0.07a	5.29 ± 0.09a,b	5.12 ± 0.02b	5.12 ± 0.02b	97.37 ± 0.81b
3	5.62 ± 0.05a,b	5.26 ± 0.05b,c	5.17 ± 0.03c	5.13 ± 0.03c	94.27 ± 2.17c
4	5.53 ± 0.08b,c	5.14 ± 0.08c	5.06 ± 0.10c	5.03 ± 0.10c	90.47 ± 0.71d
5	5.26 ± 0.06d,e	5.09 ± 0.03e	5.03 ± 0.02e	5.03 ± 0.02e	87.7 ± 1.21e

*Note:* The letters a, b, c, and d indicate significance difference (*p* < 0.05) in pH and drug content of BPG containing formulations.

**Table 8:** pH and drug content of SCMC containing metronidazole and metronidazole benzoate suspension.

**Conc.**	**SCMC containing formulation**				
	**pH**				**Drug content (%)**
	**1st day**	**7th day**	**14th day**	**21th day**	**1st day**
1	5.15 ± 0.11a	5.22 ± 0.09a	5.31 ± 0.12a	5.31 ± 0.12a	102.77 ± 2.12a
2	5.35 ± 0.17a,b	5.41 ± 0.06a,b	5.55 ± 0.14a	5.55 ± 0.14a	97.77 ± 1.82b
3	5.46 ± 0.15a,b	5.65 ± 0.13b	5.75 ± 0.13a	5.75 ± 0.13a	95.9 ± 1.37b
4	5.67 ± 0.12c,d	5.98 ± 0.06c	6.29 ± 0.14b	6.29 ± 0.14b	94.1 ± 1.21b,c
5	5.9 ± 0.10d	6.22 ± 0.15c	6.53 ± 0.34b	6.53 ± 0.34b	91.5 ± 0.92c

*Note:* The letters a, b, c, and d indicate significance difference (*p* < 0.05) in pH and drug content of SCMC containing formulations.

**Table 9:** pH and drug content of TG containing metronidazole benzoate suspension.

**Conc.**	**TG containing formulation**				
	**pH**				**Drug content (%)**
	**1st day**	**7th day**	**14th day**	**21th day**	**1st day**
1	6.10 ± 0.12a	6.22 ± 0.16b,c	6.70 ± 0.07c	6.70 ± 0.07c	97.22 ± 1.00a
2	6.35 ± 0.71b,c	6.41 ± 0.17c,d	6.75 ± 0.18d	6.75 ± 0.18d	93.40 ± 1.20b
3	6.46 ± 0.52b,c	6.65 ± 0.11c,d	6.80 ± 0.42d	6.80 ± 0.42d	93.00 ± 1.00b
4	6.67 ± 0.22c,d	6.72 ± 0.15d,e	6.87 ± 0.22e	6.87 ± 0.22e	90.5 ± 1.10c
5	6.86 ± 0.14e,f	6.91 ± 0.06f,g	6.98 ± 0.10g	6.98 ± 0.10g	88.6 ± 0.80d

*Note:* The letters a, b, c, d, e, f, and g indicate the significance difference (*p* < 0.05) in pH and drug content of TG containing formulations.

## Data Availability

All relevant data are within the paper. However, the data used to support the findings of this study are available from the corresponding author upon request.

## Nomenclature

ANOVA: analysis of variance
BPG: *Boswellia papyrifera* gum
FB: formulations containing *Boswellia papyrifera* gum
FS: formulations containing sodium carboxy methyl cellulose
FT: formulations containing tragacanth gum
HCL: hydrochloric acid
NaCl: sodium chloride
NaOH: sodium hydroxide
pH: power of hydrogen
RPM: revolution per minute
*SCMC*: sodium carboxy methyl cellulose
SD: standard deviation
TG: tragacanth gum
UV: ultraviolet radiation
